# Assessment of various efficacy outcomes using ERIVANCE-like criteria in patients with locally advanced basal cell carcinoma receiving sonidegib: results from a preplanned sensitivity analysis

**DOI:** 10.1186/s12885-021-08968-1

**Published:** 2021-11-19

**Authors:** Ralf Gutzmer, Caroline Robert, Carmen Loquai, Dirk Schadendorf, Nicholas Squittieri, Ramon Arntz, Serena Martelli, Reinhard Dummer

**Affiliations:** 1 Department Dermatology, Johannes Wesling Hospital Minden, Ruhr-University Bochum, Hans-Nolte-Str. 1, 32429 Minden, Germany; 2 Department of Dermatology, Institut Gustave Roussy and Paris-Saclay University, Villejuif, France; 3 Department of Dermatology, University Medical Center Mainz, Mainz, Germany; 4 Dermatology Clinic, University Hospital, Essen, Germany; 5 German Cancer Consortium, Heidelberg, Germany; 6 Medical Affairs Oncology, Sun Pharmaceutical Industries, Inc., Princeton, NJ USA; 7 Sun Pharmaceutical Industries, (Europe) B.V., Hoofddorp, North Holland Netherlands; 8 Skin Cancer Center University Hospital, Zürich, Switzerland

**Keywords:** Basal cell carcinoma, Hedgehog pathway inhibitor, Sonidegib, mRECIST, Tumor outcome

## Abstract

**Background:**

The BOLT study for sonidegib, a Hedgehog pathway inhibitor (HHI) approved for patients with locally advanced basal cell carcinoma (laBCC) not amenable to curative surgery or radiotherapy, used modified Response Evaluation Criteria in Solid Tumors (mRECIST) for laBCC tumor evaluation. The ERIVANCE study for vismodegib, another HHI, used a composite RECIST endpoint of ≥30% reduction in externally visible tumor or radiographic dimension, or complete ulceration resolution. This preplanned sensitivity BOLT analysis evaluated efficacy outcomes using ERIVANCE-like criteria in patients with laBCC who received sonidegib 200 mg once daily.

**Methods:**

This phase 2, double-blind study randomized patients 1:2 to sonidegib 200:800 mg daily, respectively. Key endpoints included objective response rate (ORR), duration of response (DOR), complete response (CR), partial response (PR), stable disease (SD), and progressive disease (PD). laBCC tumors were assessed by both mRECIST and ERIVANCE-like criteria. Per mRECIST, an overall response of CR was based on negative histology; photographic assessment of CR, PR (scar/fibrosis only), SD (scar/fibrosis only), or not available (NA); and a magnetic resonance imaging response of CR or NA. An overall response of CR was primarily based on negative histology using ERIVANCE-like criteria.

**Results:**

Per mRECIST criteria, ORR (95% confidence interval [CI]) by central and investigator review for patients with laBCC (*n* = 66) was 56.1% (43.3–68.3%) and 71.2% (58.7–81.7%), respectively. CR per central review was achieved in 3 (4.5%) patients and PR, SD, and PD occurred in 34 (51.5%), 23 (34.8%), and 1 (1.5%) patient, respectively. Median (95% CI) DOR was 26.1 months (not estimable [NE]).

Using ERIVANCE-like criteria, efficacy outcomes per central and investigator review were higher, with an ORR (95% CI) of 60.6% (47.8–72.4%) and 74.2% (62.0–84.2%), respectively. CR per central review was reached in 14 (21.2%) patients and PR, SD, and PD occurred in 26 (39.4%), 20 (30.3%), and 1 (1.5%) patient, respectively. DOR was unchanged with a median (95% CI) of 26.1 months (NE).

**Conclusions:**

Overall, applying ERIVANCE-like criteria to patients with laBCC receiving sonidegib 200 mg daily yielded higher response rates vs mRECIST criteria.

**Trial registration:**

BOLT registration: ClinicalTrials.gov (NCT01327053) on March 30, 2011.

**Supplementary Information:**

The online version contains supplementary material available at 10.1186/s12885-021-08968-1.

## Background

Basal cell carcinoma (BCC) is the most common cancer worldwide and constitutes 80% of the 3.5 million cases of nonmelanoma skin cancer diagnosed each year [1–3]. In most cases of BCC, the American Academy of Dermatology and the European consensus-based interdisciplinary guidelines recommend surgery as the standard treatment, which has an excellent prognosis [4–6]. However, in cases of advanced BCC, treatment options are limited due to significant tissue invasion and associated morbidity [2, 7]. Advanced BCC can be characterized as either locally advanced BCC (laBCC), in which tumors are substantially enlarged and locally destructive to the point that surgery is no longer feasible or surgical resection would result in considerable deformity; or metastatic BCC (mBCC), which is associated with high morbidity and mortality [7]. In an analysis at a tertiary referral center, the prevalence of complicated BCC cases was estimated to be 7.2% of all BCC cases; these patients generally also had greater variations of BCC histologic subtypes compared with patients with non-advanced cases of BCC [8]. Moreover, patients with advanced BCC were more likely to have histologically aggressive subtypes of BCC compared with patients with non-advanced BCC, which increases the risk of tumor recurrence due to a greater likelihood of incomplete primary excision [8, 9]. Consequently, patients with advanced BCC may have complications and require extensive and prolonged treatment.

Development of BCC is associated with upregulation of the Hedgehog signaling pathway, with somatic mutations of this pathway present in most cases of BCC [1, 7, 10]. Consequently, in patients with advanced BCC, when surgery and radiotherapy are contraindicated or pose a significant management challenge, such as substantial posttreatment deformity, systemic Hedgehog pathway inhibitors (HHIs) are recommended [4, 6, 7]. Two HHIs are currently approved for the treatment of advanced BCC: sonidegib (Odomzo®; Sun Pharmaceutical Industries, Inc.; Cranbury, NJ) and vismodegib (Erivedge®; Genentech, Inc.; San Francisco, CA), which both target selectively Smoothened, a Hedgehog pathway protein [11, 12]. Sonidegib is approved in the US, EU, Switzerland, and Australia for the treatment of adult patients with laBCC  who are not amenable to curative surgery or radiation therapy [11, 13–15]. Additionally, sonidegib is approved to treat mBCC in Switzerland and Australia [14, 15]. Vismodegib is approved in the US for the treatment of adults with mBCC or laBCC that has recurred after surgery, or for those who are not surgical or radiotherapy candidates, and is approved in the EU, Switzerland, and Australia for adults with laBCC or mBCC when surgery or radiotherapy is inappropriate [12, 16–18].

In the pivotal 42-month phase 2 BOLT (Basal Cell Carcinoma Outcomes with LDE225 [sonidegib] Treatment) study (NCT01327053) evaluating efficacy and safety in patients with laBCC and mBCC, sonidegib 200 mg once daily (QD) demonstrated robust efficacy and manageable toxicity [19–22]. Tumor assessments for patients with laBCC were performed by a central review committee and investigators using modified Response Evaluation Criteria in Solid Tumors (mRECIST). These modified criteria were used in place of RECIST v1.1 criteria to address the challenges in determining tumor response in patients with laBCC due to potential morphological changes following treatment, including ulceration, cyst formation, fibrosis, and poorly defined lesions [22]. In contrast, in the phase 2, single-arm, 2-cohort ERIVANCE study evaluating efficacy and safety of vismodegib, tumor response in patients with laBCC was assessed using a composite endpoint integrating a ≥ 30% reduction into the externally visible or radiographic dimension of the tumor (RECIST criteria) or complete ulceration resolution if present at baseline [23, 24].

Overall, both the mRECIST criteria used in the BOLT study and the composite RECIST criteria used in the ERIVANCE study incorporate magnetic resonance imaging (MRI) per RECIST v1.1, color photography per World Health Organization (WHO) guidelines, and histology for laBCC tumor assessment. However, the algorithm determining overall response differs between the two criteria used, and mRECIST utilizes more stringent methods of tumor assessment to calculate a composite overall response, resulting in efficacy outcomes for which the response is lower for mRECIST compared with RECIST v1.1 [21]. In the absence of head-to-head clinical studies comparing sonidegib and vismodegib, a preplanned sensitivity analysis in the BOLT trial, which applied ERIVANCE-like criteria to laBCC tumor outcomes, was performed. Here, we present the results of this sensitivity analysis evaluating the subset population of patients with laBCC who received the approved 200 mg QD dose of sonidegib.

## Methods

### Study design

BOLT was a multicenter, double-blind, randomized, phase 2 international clinical study, and enrolled patients were randomized 1:2 to sonidegib 200 or 800 mg QD. Patient baseline clinical characteristics and study design were previously described [19–22]. Patients eligible for inclusion in the study were ≥18 years of age and had either histologically confirmed laBCC that was not amenable to curative surgery, radiotherapy, or localized therapies, or histologically confirmed mBCC. Patients with laBCC were required to have measurable disease, defined as ≥1 lesion that could be accurately measured in at least one dimension as ≥10 mm with an MRI scan or with annotated color photographs. Patients additionally needed a WHO performance status of ≤2 and sufficient bone marrow and liver and renal function. At screening, punch tumor biopsies within 21 days prior to the initiation of study treatment were required for all patients with accessible laBCC tumors. For patients without accessible laBCC tumors, such as tumors in difficult anatomical locations, collection of an archival tumor tissue sample was required at screening.

The primary endpoint was objective response rate (ORR) per central review, defined as the percentage of patients with a best overall response of complete response (CR) or partial response (PR) based on consecutive evaluations ≥4 weeks apart. Secondary assessments included CR, PR, stable disease (SD), progressive disease (PD), and duration of response (DOR). laBCC tumors were assessed utilizing mRECIST criteria integrating MRI, color photography, and histological tumor tissue evaluation and then analyzed in a preplanned sensitivity analysis with criteria similar to that used in the ERIVANCE study.

Per mRECIST criteria, an overall composite response of CR was based on the following measures: negative histology for multiple punch biopsies; photographic assessment of CR, PR (scar/fibrosis only), SD (scar/fibrosis only), or not available (NA); and an MRI response of either CR or NA (Table 1). A minimum of two 3–4 mm punch biopsies were required per patient, although the specific number of biopsy samples taken depended on the size of the original target tumor with approximately one 3–4 mm sample taken from every 4-cm^2^ area of tumor tissue. PR per mRECIST was determined by the following: negative histology, CR, PR (scar/fibrosis only), or SD (scar/fibrosis only) per photographic assessment, and an MRI response of either PR or SD; negative histology, PR per photographic assessment, and MRI response of CR, PR, SD, or NA; or, alternatively, negative histology, NA per photographic assessment, and an MRI response of PR. Per the ERIVANCE-like criteria, CR was based on negative histology and CR, PR, SD, or NA for MRI assessment and photographic evaluation. In addition, PR per ERIVANCE-like criteria was defined as histological assessment of positive or unknown, SD or SD (scar/fibrosis only) per photographic evaluation, and an MRI response of CR or PR. This ERIVANCE-like criteria used to define PR were the same as the mRECIST-defining criteria for SD. Consequently, the criteria used to define CR per ERIVANCE-like criteria were often the same as those used to define PR per mRECIST.

**Table 1:** Composite overall response in laBCC determined by ERIVANCE-like vs mRECIST criteria

MRI^a^	Photograph^b^	Histology^c^	Composite overall response	
			mRECIST^d,e^	ERIVANCE-like criteria
CR	CR	Negative	CR^c^	CR
	PR (scar/fibrosis only) or SD (scar/fibrosis only)	Negative		
	NA	Negative		
NA	CR	Negative	CR^c^	CR
	PR (scar/fibrosis only) or SD (scar/fibrosis only)	Negative		
PR	CR	Negative	PR	CR
	PR (scar/fibrosis only) or SD (scar/fibrosis only)	Negative		
SD	CR	Negative	PR	CR
	PR (scar/fibrosis only) or SD (scar/fibrosis only)	Negative		
CR	PR	Negative	PR	CR
PR				
SD				
NA				
CR	SD	Negative	SD	CR
PR				
PR	NA	Negative	PR	CR
CR	SD	Positive or unknown	SD	PR
	SD (scarring/fibrosis only)			
PR	SD	Positive or unknown	SD	PR
	SD (scarring/fibrosis only)			

^a^Measurability per central review per RECIST v1.1 [23]

^b^PR ≥50% reduction in the sum of perpendicular products; PD ≥25% increase in the sum of products per WHO criteria [25]

^c^Confirmed CRs required multiple punch biopsy samples per lesion

^d^An independent review committee reevaluated all assessments for the laBCC cohort to determine a composite response

^e^Since posttreatment ulceration, cyst formation, and scarring/fibrosis may be considered treatment effects and are not necessarily indicative of disease progression in laBCC, “scarring/fibrosis only” was allowed per mRECIST criteria, given that the other measures such as histology and MRI also showed no signs of disease progression

*CR* complete response, *laBCC* locally advanced basal cell carcinoma, *mRECIST* modified RECIST, *MRI* magnetic resonance imaging, *NA* not available, *PD* progressive disease, *PR* partial response, *RECIST* Response Evaluation Criteria in Solid Tumors, *SD* stable disease, *WHO* World Health Organization

Written informed consent was obtained from all patients prior to the start of the study. The study protocol and all amendments were approved by the independent ethics committee or institutional review board for each center (Supplementary Table 1). This study was carried out in accordance with the ethical principles of the Declaration of Helsinki.

### Assessments

All patients followed the same treatment protocol of an oral dose of sonidegib 200 or 800 mg QD until PD (determined by radiological scans or clinical photography per central review), intolerable toxicity, withdrawal of consent, study discontinuation, or death. Only data for patients with laBCC receiving the approved 200 mg QD dose are reported in this analysis. Following baseline tumor assessments at screening, additional tumor response evaluations were performed at weeks 5, 9, and 17 (±3 days) and subsequently every 8 weeks (±3 days) during the first year, followed by every 12 weeks (±3 days) thereafter until confirmation of PD, start of a new antineoplastic therapy, loss to follow-up, or end of treatment.

Safety assessments involved monitoring and recording adverse events (AEs), including serious AEs, in addition to regular monitoring of hematology, clinical chemistry laboratory, and electrocardiogram abnormalities, as well as checking vital signs and physical condition.

### Statistical analyses

All statistical methods were previously described in detail [19–22]. Treatment with sonidegib was considered efficacious when the ORR of any treatment arm at the end of the study was ≥30%. Primary and secondary endpoints were estimated with 95% confidence intervals (CIs), with a lower bound of the 95% CI exceeding 20% considered clinically meaningful. ORR was calculated using point estimates and 95% CI, while medians and 95% CIs were derived using Kaplan-Meier methodology for DOR evaluations.

## Results

### Baseline patient characteristics

Among the 230 patients enrolled in BOLT, 66 patients with laBCC were randomized to sonidegib 200 mg. Patient demographics and baseline disease characteristics were reported previously (Supplementary Table 2) [22]. Of the patients with laBCC receiving sonidegib 200 mg QD, 37 (46.8%) had aggressive and 29 (36.7%) had nonaggressive subtypes. Overall, median duration of exposure to sonidegib was 11 months for all patients (laBCC and mBCC combined) receiving sonidegib 200 mg; 92.4% of all patients in the sonidegib 200 mg arm discontinued treatment by the end of the study. The most common reasons for study discontinuation for all patients in the sonidegib 200 mg arm were PD (36.7%), AEs (29.1%), physician decision (12.7%), patient decision (10.1%), loss to follow-up (2.5%), and death (1.3%).

### Per central review

#### Best overall response using ERIVANCE-like vs mRECIST criteria

Through 42 months of treatment with sonidegib 200 mg QD, ORR (95% CI) for patients with laBCC was 56.1% (43.3–68.3%) per mRECIST (Table 2). CR was achieved in 3 (4.5%) patients, while PR occurred in 34 (51.5%) patients per central review. Comparatively, when using ERIVANCE-like criteria, ORR (95% CI) per central review was 60.6% (47.8–72.4%) for patients with laBCC receiving sonidegib 200 mg QD. Similarly, CR was achieved in 14 (21.2%) patients and PR occurred in 26 (39.4%) patients.

**Table 2:** Best overall response in patients with laBCC receiving sonidegib 200 mg daily by central review

	mRECIST criteria	ERIVANCE-like criteria
**laBCC, all**	*n* = 66	*n* = 66
ORR, % (95% CI)	56.1 (43.3–68.3)	60.6 (47.8–72.4)
CR, % (95% CI)	4.5 (0.9–12.7)	21.2 (12.1–33.0)
PR, %	51.5	39.4
SD, %	34.8	30.3
PD, %	1.5	1.5
Unknown, %	7.6	7.6
**laBCC, aggressive histology**^**a**^	*n* = 37	*n* = 37
ORR, % (95% CI)	59.5 (42.1–75.2)	64.9 (47.5–79.8)
CR, % (95% CI)	5.4 (0.7–18.2)	21.6 (9.8–38.2)
PR, %	54.1	43.2
SD, %	32.4	27.0
PD, %	2.7	2.7
Unknown, %	5.4	5.4
**laBCC, nonaggressive histology**^**b**^	*n* = 29	*n* = 29
ORR, % (95% CI)	51.7 (32.5–70.6)	55.2 (35.7–73.6)
CR, % (95% CI)	3.4 (0.1–17.8)	20.7 (8.0–39.7)
PR, %	48.3	34.5
SD, %	37.9	34.5
PD, %	0	0
Unknown, %	10.3	10.3

^a^Includes basosquamous, micronodular infiltrative, multifocal, and sclerosing histological subtypes

^b^Includes nodular and superficial histological subtypes

*CI* confidence interval, *CR* complete response, *laBCC* locally advanced basal cell carcinoma, *mRECIST* modified RECIST, *ORR* overall response rate, *PD* progressive disease, *PR* partial response, *RECIST* Response Evaluation Criteria in Solid Tumors, *SD* stable disease

When tumor response was analyzed by histologic subtype, ORR (95% CI) per mRECIST for patients with aggressive vs nonaggressive subtypes of laBCC was 59.5% (42.1–75.2%) vs 51.7% (32.5–70.6%), respectively. CR per mRECIST was achieved in 2 (5.4%) patients and 1 (3.4%) patient with aggressive and nonaggressive subtypes, respectively. In contrast, ORR (95% CI) per ERIVANCE-like criteria for patients with aggressive vs nonaggressive histologic subtypes was 64.9% (47.5–79.8%) vs 55.2% (35.7–73.6%), respectively. Moreover, CR per ERIVANCE-like criteria was achieved in 8 (21.6%) patients and 6 (20.7%) patients with aggressive and nonaggressive subtypes, respectively.

#### Duration of response using ERIVANCE-like vs mRECIST criteria

In patients with laBCC receiving sonidegib 200 mg, median (95% CI) DOR was 26.1 months (not estimable [NE]) with event-free probability estimates (95% CI) for 12 months of 64.9% (42.3–80.4%), per mRECIST (Table 3). When DOR was assessed using ERIVANCE-like criteria, it was unchanged with a median DOR (95% CI) of 26.1 months (NE). However, event-free probability estimates (95% CI) were higher for 12 months (69.2% [46.5–83.8%]).

**Table 3:** Duration of response in patients with laBCC receiving sonidegib 200 mg daily by central review

	mRECIST criteria	ERIVANCE-like criteria
**DOR**, median, months, (95% CI)	26.1 (NE)	26.1 (NE)
**Event-free probability estimate**, %, (95% CI)		
6 months	86.4 (67.7–94.7)	90.8 (74.1–96.9)
9 months	74.9 (54.4–87.2)	83.8 (65.3–93.0)
12 months	64.9 (42.3–80.4)	69.2 (46.5–83.8)

*CI* confidence interval, *DOR* duration of response, *laBCC* locally advanced basal cell carcinoma, *mRECIST* modified RECIST, *NE* not estimable, *RECIST* Response Evaluation Criteria in Solid Tumors

Analyses of event-free probability estimates by histologic subtype demonstrated overall higher estimates for 6, 9, and 12 months per ERIVANCE-like criteria vs mRECIST for patients with nonaggressive subtypes of laBCC (92.3% [56.6–98.9%] vs 91.7% [53.9–98.8%], 84.6% [51.2–95.9%] vs 75.0% [40.8–91.2%], and 70.5% [30.6–90.2%] vs 75.0% [40.8–91.2%], respectively. Similarly, event-free probability estimates were greater for patients with aggressive subtypes per ERIVANCE-like criteria vs mRECIST with 6-, 9-, and 12-month estimates of 89.9% (65.3–97.4%) vs 82.9% (55.7–94.2%), 83.5% (56.7–94.5%) vs 76.0% (47.6%–90.3), and 67.5% (37.3–85.5%) vs 57.9% (27.6–79.3%), respectively.

### Per investigator review

#### Best overall response using ERIVANCE-like vs mRECIST criteria

Analyses of tumor response by investigator review showed greater responses per ERIVANCE-like criteria vs mRECIST for patients with laBCC with an ORR (95% CI) of 74.2% (62.0–84.2%) vs 71.2% (58.7–81.7%). Per mRECIST, CR was achieved in 6 (9.1%) patients, while PR occurred in 41 (62.1%) patients per investigator review (Table 4). In contrast, CR was achieved in 19 (28.8%) patients and PR occurred in 30 (45.5%) patients using ERIVANCE-like criteria.

**Table 4:** Best overall response in patients with laBCC receiving sonidegib 200 mg daily by investigator review

	mRECIST criteria	ERIVANCE-like criteria
**laBCC, all**	*n* = 66	*n* = 66
ORR, % (95% CI)	71.2 (58.7–81.7)	74.2 (62.0–84.2)
CR, % (95% CI)	9.1 (3.4–18.7)	28.8 (18.3–41.3)
PR, %	62.1	45.5
SD, %	19.7	16.7
PD, %	1.5	1.5
Unknown, %	7.6	7.6
**laBCC, aggressive histology**^**a**^	*n* = 37	*n* = 37
ORR, % (95% CI)	70.3 (53.0–84.1)	75.7 (58.8–88.2)
CR, % (95% CI)	8.1 (1.7–21.9)	29.7 (15.9–47.0)
PR, %	62.2	45.9
SD, %	21.6	16.2
PD, %	0	0
Unknown, %	8.1	8.1
**laBCC, nonaggressive histology**^**b**^	*n* = 29	*n* = 29
ORR, % (95% CI)	72.4 (52.8–87.3)	72.4 (52.8–87.3)
CR, % (95% CI)	10.3 (2.2–27.4)	27.6 (12.7–47.2)
PR, %	62.1	44.8
SD, %	17.2	17.2
PD, %	3.4	3.4
Unknown, %	6.9	6.9

^a^Includes basosquamous, micronodular infiltrative, multifocal, and sclerosing histological subtypes

^b^Includes nodular and superficial histological subtypes

*CI* confidence interval, *CR* complete response, *laBCC* locally advanced basal cell carcinoma, *mRECIST* modified RECIST, *ORR* overall response rate, *PD* progressive disease, *PR* partial response, *RECIST* Response Evaluation Criteria in Solid Tumors, *SD* stable disease

When tumor response was analyzed by histologic subtype, ORR (95% CI) per mRECIST for patients with aggressive vs nonaggressive subtypes of laBCC was 70.3% (53.0–84.1%) vs 72.4% (52.8–87.3%), respectively. Conversely, ORR (95% CI) using ERIVANCE-like criteria for patients with aggressive vs nonaggressive subtypes of laBCC was 75.7% (58.8–88.2%) vs 72.4% (52.8–87.3%), respectively.

#### Duration of response using ERIVANCE-like vs mRECIST criteria

Median DOR (95% CI) using mRECIST criteria per investigator review for patients with laBCC receiving sonidegib 200 mg QD was 15.7 (12.0–20.2) months compared with a median DOR of 18.2 (12.9–24.0) months using ERIVANCE-like criteria. Event-free probability estimates using mRECIST criteria for 6, 9, and 12 months were 89.8% (74.8–96.1%), 80.7% (63.5–90.4%), and 71.4% (53.1–83.6%), respectively. In contrast, event-free probability estimates using ERIVANCE-like criteria were higher for 6, 9, and 12 months at 93.2% (75.5–98.3%), 89.2% (69.9–96.4%), and 84.7% (63.9–94.0%), respectively.

### Safety

As previously described, the majority of AEs were manageable and consistent with prior assessments at the final 42-month BOLT analysis [19]. In patients receiving sonidegib 200 mg QD, AEs were predominantly Grade 1 or 2, and the incidence of serious AEs considered related to treatment was observed in 4 (5.1%) patients. AEs reported in ≥30% of patients receiving sonidegib 200 mg QD were muscle spasms (54.4%), alopecia (49.4%), dysgeusia (44.3%), nausea (39.2%), fatigue (32.9%), diarrhea (31.6%), elevated blood creatine phosphokinase (30.4%), and weight loss (30.4%).

## Discussion

This preplanned sensitivity analysis of the BOLT study demonstrated greater tumor response rates when applying ERIVANCE-like criteria compared with mRECIST criteria for patients with laBCC receiving sonidegib 200 mg QD. Overall, ORRs per central and investigator reviews were higher with ERIVANCE-like criteria vs mRECIST. In particular, the number of patients who achieved CR following treatment was substantially higher when ERIVANCE-like criteria were applied vs mRECIST—highlighting the differences in how tumor response and CR were defined between the two criteria sets. Additionally, tumor responses considered PR per mRECIST were defined as CR with ERIVANCE-like criteria, and patients with SD per mRECIST were considered either CR or PR when ERIVANCE-like criteria were applied. In particular, the most substantial difference between the two criteria sets is that when using the ERIVANCE-like criteria, patients with negative histology were considered CR regardless of their response based on MRI or color photography. In contrast, the mRECIST criteria required patients to have photographic assessment of CR, PR (scar/fibrosis only), SD (scar/fibrosis only), or NA and an MRI response of either CR or NA, in addition to negative histology for a patient to be considered to have achieved an overall composite response of CR.

These variations in how tumor response is assessed in patients with laBCC may stem from a lack of standardized terminology to define laBCC. Currently, the definition of laBCC can be grouped within the broader and vague terminology used to describe advanced BBC—which evolved for patients with BCC who were not surgical or radiotherapy candidates—and indicates patients who have a long disease history without treatment, were refractory to treatment, or had disease recurrence following treatment [6]. In addition, these patients may have widespread tissue destruction in the surrounding tumor area and curative resection with surgery or radiotherapy is no longer feasible or would result in significant deformity [6]. Moreover, common terms used to describe advanced BCC such as “locally advanced,” “inoperable,” and “considerable morbidity or deformity post-surgery” are subjective and highly dependent on the managing physician [4]. Consequently, the varying challenges in describing and managing advanced BCC result in a generalized and ambiguous definition of laBCC.

Furthermore, unlike other solid tumors, BCC does not have a standard staging system due to the heterogenous nature and development of these tumors, which do not always follow the typical 3-step tumor-node-metastasis process (eg, tumor, nodal development, and distant metastases) [6]. As a result, progression-free survival (PFS) and overall survival results are less meaningful, and outcomes are not able to be accurately measured with RECIST criteria, as extensive tissue damage can occur without affecting survival in patients with laBCC [6]. Accordingly, in the ERIVANCE clinical studies evaluating safety and efficacy of vismodegib, a composite endpoint was designed to assess tumor response in patients with laBCC, since there was no standardized endpoint for laBCC [24, 26]. Specifically, CR was defined as an absence of residual BCC in a biopsy sample, and PR was defined as a reduction of ≥30% in the radiographic or externally visible diameter of the tumor, or complete resolution of ulceration (if present at baseline) [26].PD was defined as an increase of ≥20% in radiographic or externally visible tumor dimension, development of new ulceration, or the presence of new lesions [24, 26]. Similarly, in the BOLT study, a composite endpoint defined by mRECIST criteria evaluated tumor response in patients with laBCC to address the challenges and shortcomings of RECIST to accurately measure efficacy in these patients [22]. Consequently, differences in the specific endpoints and criteria used to define outcomes such as ORR, CR, PR, SD, and PD for the BOLT and ERIVANCE studies result in efficacy outcomes that are difficult to compare for patients with laBCC. Hence, this preplanned sensitivity analysis applying ERIVANCE-like criteria to the BOLT outcomes was performed to generate more comparative results for sonidegib vs vismodegib. Subsequently, the results presented in this analysis demonstrate the differences used to evaluate tumor response  and yielded higher response rates when using ERIVANCE-like criteria compared with the mRECIST criteria used in BOLT.

Limitations of this sensitivity analysis include the lack of PFS and time to tumor response data using ERIVANCE-like criteria in patients with laBCC. In addition, the efficacy of sonidegib in patients with recurrent disease following previous therapy with an HHI is unknown, since these patients were excluded from the BOLT study [21].

Overall, when ERIVANCE-like criteria were applied to patients with laBCC receiving sonidegib 200 mg QD, tumor response rates were higher compared with the analyses using mRECIST criteria. These results continue to support the use of sonidegib to treat patients with laBCC. Since there are no current standardized assessment criteria for laBCC, practitioners should be cognizant of the specific differences between the two sets of criteria used to evaluate sonidegib and vismodegib when choosing a treatment regimen for their patients. Future studies on advanced BCC would benefit from a standardized definition of laBCC and uniform criteria to evaluate tumor response to better enable more accurate comparisons of efficacy across treatment options.

## Supplementary Information

**Additional file 1.**

## Data Availability

The datasets created and analyzed during this study are available from the corresponding author upon reasonable request.

## Abbreviations

AE: adverse event
BCC: basal cell carcinoma
BOLT: Basal Cell Carcinoma Outcomes with LDE225 [sonidegib] Treatment
CR: complete response
DOR: duration of response
HHI: Hedgehog pathway inhibitor
laBCC: locally advanced BCC
mBCC: metastatic BCC
mRECIST: modified Response Evaluation Criteria in Solid Tumors
MRI: magnetic resonance imaging
NE: not estimable
ORR: objective response rate
PD: progressive disease
PFS: progression-free survival
PR: partial response
QD: once daily
RECIST: Response Evaluation Criteria in Solid Tumors
SD: stable disease
WHO: World Health Organization
