# Supplementary material for: Assessment of various efficacy outcomes using ERIVANCE-like criteria in patients with locally advanced basal cell carcinoma receiving sonidegib: results from a preplanned sensitivity analysis
Source: BMC Cancer. 2021 Nov 19;21:1244. doi: 10.1186/s12885-021-08968-1 (PMC8603490; doi:10.1186/s12885-021-08968-1)
Supplement: Supplementary file 1 — Additional file 1. [file 12885_2021_8968_MOESM1_ESM.docx]

**Supplementary material**

**Supplementary table 1.** List of Independent Ethics Committees and Institutional Review Boards

| **IEC or IRB** | **Department/Organization** | **City, Country** |
| --- | --- | --- |
| Northern Sydney Coast Human Research Ethics Committee | Research Office | St Leonards, Australia |
| Commission d’ethique UCL Saint-Luc | Avenue Hippocrate 10 | Brussels, Belgium |
| Ethisch comité GZA Sint-Augustinus | Oosterveldlaan 24 | Wilrijk, Belgium |
| CPP lle de France IV-SAINT LOUIS | — | Paris, France |
| Ethik-Kommission der Medizinischen Hochschule Hannover | — | Hannover, Germany |
| Landesärztekammer Rheinland-Pfalz | Ethik-Kommission | Mainz, Germany |
| Ludwig-Maximilians Universität München Klinikum der Universität | Ethik-Kommission | Muenchen, Germany |
| Landesärztekammer Thüringen | Ethik-Kommission | Jena-Maua, Germany |
| Ethikkommission der Ärztekammer Westfalen-Lippe und der Medizinischen Fakultät der Westfälischen Wilhelms-Universität Münster | — | Muenster, Germany |
| Ethik-Kommission der Medizinischen Fakultät der Christian-Albrechts-Universität zu Kiel | — | Kiel, Germany |
| Universitätsklinikum Essen Medizinische Fakultät der Universität Duisburg-Essen | Ethik-Kommission | Essen, Germany |
| Landesamt für Gesundheit und Soziale | Geschäftsstelle der Ethik-  Kommission des Landes  Berlin | Berlin, Germany |
| Albert-Ludwigs-Universität Freiburg | Ethik-Kommission | Freiburg, Germany |
| Ärztekammer Niedersachsen | Ethik-Kommission | Hannover, Germany |
| Tecnico-Scientifica Comitato Etico Interaziendale | — | Torino, Italy |
| Vall d'Hebron Institut de Recerca | — | Barcelona, Spain |
| CEIC Area 5-Hospital  Universitario La Paz | — | Madrid, Spain |
| Comite Etico de Investigaction Clinica Hospital Ramon y Cajal | — | Madrid, Spain |
| Kantonale Ethikkommission Zurich | — | Zurich, Switzerland |
| Kantonale Ethikkommission Bern | — | Bern, Switzerland |
| Commission d'ethique de la recherche sur letre humain | — | Geneva, Switzerland |
| NRES Committee Yorkshire & The Humber-Sheffield | — | Leeds, UK |
| IRB Services | — | Aurora, Canada |
| Northwestern University | Office for the Protection of  Research Subjects | Chicago, USA |
| Western Institutional Review Board | — | Olympia, USA |
| The University of Texas, MD Anderson Cancer Center | — | Houston, USA |
| Office for the Protection of Research Subjects | — | Los Angeles, USA |
| Quorum | — | Seattle, USA |
| Penn State College of Medicine | — | Hershey, USA |
| Medstar Health Research Institute-Georgetown University | Oncology Institutional  Review Board | Washington DC, USA |
| Stanford University | Research Compliance Office | Palo Alto, USA |
| Human Research Protection Office | — | St Louis, USA |
| NYU School of Medicine | — | New York, USA |
| Office of Human Research Studies Dana Farber Cancer Institute | — | Boston, USA |
| University of Utah Institutional Review Board | — | Salt Lake City, USA |
| US Oncology Inc. | Institutional Review Board | The Woodlands, USA |
| Scientific Review Board of University Hospital “Andreas Syggros” | — | Athens, Greece |
| Medical Research Council Ethics Committee for Clinical Pharmacology | — | Budapest, Hungary |

IEC, Independent Ethics Committee; IRB, Institutional Review Board; UK, United Kingdom; USA, United States of America.

**Supplementary table 2.** Baseline demographics and disease characteristics in patients receiving sonidegib 200 mg daily [20, 23]

|  | **All patients**  **(n = 79)** |
| --- | --- |
| **Age**, years, median (range) | 67 (25–92) |
| **Sex,** male | 48 (60.8) |
| **ECOG performance status** |  |
| 0 | 50 (63.3) |
| 1 | 19 (24.1) |
| 2 | 8 (10.1) |
| Unknown | 2 (2.5) |
| **Stage** |  |
| laBCC | 66 (83.5) |
| mBCC | 13 (16.5) |
| **Histologic/cytologic subtype** |  |
| Aggressive^a^ | 40 (50.6) |
| Nonaggressive^b^ | 38 (48.1) |
| Undetermined | 1 (1.3) |
| **Number of lesions** |  |
| 0 | 0 |
| 1 | 30 (38.0) |
| ≥2 | 49 (62.0) |
| **Prior antineoplastic therapy** |  |
| Surgery | 59 (74.7) |
| Radiotherapy | 19 (24.1) |

Data presented as n (%) of patients unless otherwise indicated.

^a^Includes micronodular, infiltrative, multifocal, basosquamous, and sclerosing histological subtypes.

^b^Includes nodular and superficial histological subtypes.

BCC, basal cell carcinoma; ECOG, Eastern Cooperative Oncology Group; laBCC, locally advanced BCC; mBCC, metastatic BCC.
